# Surface Interactions between an Eco-Friendly Antifouling Agent and *Pseudoalteromonas tunicata* Membrane

**DOI:** 10.1021/acsabm.5c02341

**Published:** 2026-03-06

**Authors:** Ana Sara Gomes, Cláudia Nunes, Rita Teixeira-Santos, Maria Romeu, Maria Laura Alfieri, Sara M. M. Cravo, Filipe Mergulhão, Marta Correia-da-Silva, Salette Reis

**Affiliations:** † CIIMAR/CIMAR LA, Interdisciplinary Centre of Marine and Environmental Research, 367866 University of Porto, Terminal de Cruzeiros do Porto de Leixões, Av. General Norton de Matos, s/n, 4450-208 Matosinhos, Portugal; ‡ Laboratory of Organic and Pharmaceutical Chemistry, Faculty of Pharmacy, 386292 University of Porto, Rua Jorge de Viterbo Ferreira 228, 4050-313 Porto, Portugal; § LAQV, REQUIMTE, Departamento de Ciências Químicas, Faculdade de Farmácia, Universidade do Porto, Rua Jorge Viterbo Ferreira 228, 4050-313 Porto, Portugal; ∥ LEPABE-Laboratory for Process Engineering, Environment, Biotechnology and Energy, Faculty of Engineering, University of Porto, Rua Dr. Roberto Frias, 4200-465 Porto, Portugal; ⊥ ALiCE-Associate Laboratory in Chemical Engineering, Faculty of Engineering, University of Porto, Rua Dr. Roberto Frias, 4200-465 Porto, Portugal; # Department of Chemical Sciences, University of Naples Federico II, Via Cintia 4, I-80126 Naples, Italy

**Keywords:** Langmuir monolayer, antifouling, antibiofilm, membrane, bacteria, LPS

## Abstract

In this work, we investigated the interfacial chemistry of our recently developed antifouling polyphenolic small molecule (GBA26) directly with bacterial membrane models and the marine bacterium *Pseudoalteromonas tunicata*. Different Langmuir lipid monolayer models were constructed to mimic the interface of bacterial membranes. Surface pressure–area isotherms showed that GBA26 interacted with assembled lipid monolayers, and this interaction was influenced by the phospholipid composition. GBA26 was also able to interact with monolayers of lipopolysaccharide (LPS) extracted from *P. tunicata*. The morphology of the monolayer was also analyzed using Brewster angle microscopy (BAM), showing that GBA26 reduced lipid domains' condensation levels. Flow cytometry data indicated that GBA26 disrupts cell membranes and reduces the metabolic activity of *P. tunicata*, without inducing ROS formation. Furthermore, GBA26 significantly decreased bacterial culturability. Altogether, these results shed light on its antibiofilm mechanism against this marine bacterium.

## Graphical abstract

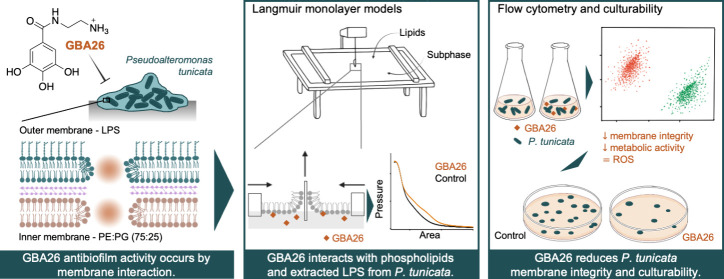

## 1 Introduction

Biofouling is the natural attachment of micro and macroorganisms to artificial submerged structures, such as docks and ship vessels. This process has detrimental effects on economic activity due to high maintenance costs and increased fuel consumption in maritime traffic caused by higher drag forces. It also impacts native biodiversity by facilitating the spread of invasive species.^1,2^ Although biocides have been used in antifouling coatings as a solution to prevent biofouling, their toxicity and bioaccumulation pose serious risks to marine ecosystems and the food chain.^3,4^ Therefore, the development of sustainable and eco-friendly antifouling agents is essential to replace current harmful biocides.

Our group has designed and synthesized a library of gallic acid derivatives,^5^ including the promising antifouling agent GBA26 (Figure 1A).^6^ Particularly, GBA26 was shown to exhibit antifouling ability by inhibiting the settlement of larvae of the macrofouler *Mytilus gallopovincialis*,^5^ and by preventing and reducing the biofilm formation of the microfouler *Pseudoalteromonas tunicata*, a marine biofilm-forming Gram-negative bacterium.^7^ Its eco-friendly profile was demonstrated as GBA26 caused no mortality in mussel larvae nor in the nontarget species *Artemia salina*. Additionally, it showed no evidence of endocrine disruption, since it did not alter the transcription of the peroxisome proliferator-activated receptor γ (PPARγ) and pregnane X receptor (PXR) nuclear receptors at low concentrations (≤10 μM).^5,7^

**Figure 1:**
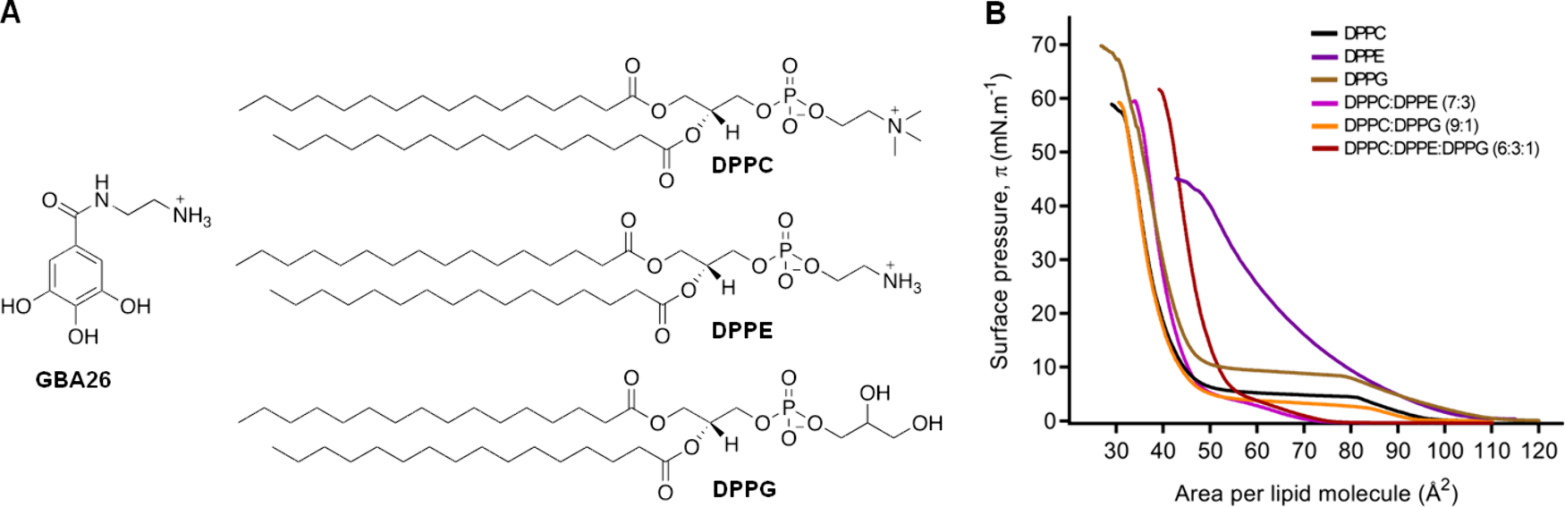
Chemical structures of GBA26 and phospholipids, and phospholipid monolayer models’ construction. (A) Antifouling compound GBA26, phosphatidylcholine (DPPC), phosphatidylethanolamine (DPPE), and phosphatidylglycerol (DPPG). (B) Surface pressure–area isotherms of monolayers consisting of DPPC (black), DPPE (purple), DPPG (brown), DPPC:DPPE (7:3) (magenta), DPPC:DPPG (9:1) (orange), and DPPC:DPPE:DPPG (6:3:1) (dark red) measured on 10 mM HEPES, 100 mM NaCl (pH 7.4) at 21 °C. Only one representative experiment of three replicates is presented per each condition.

Although several new synthetic and natural compounds with antifouling activity have been reported,^8^ comprehensive ecotoxicity assessments and elucidation of their mechanism of action are essential for safe and predictable application.^9,10^ While efforts regarding ecotoxicity have been made, few studies have addressed the molecular mechanism of action. Indeed, the discovery of effective and safe antifouling agents remains challenging, as biofouling is a complex, multiorganism and dynamic process with a multitude of potential mechanisms of action and molecular targets.^11,12^ While general antifouling mechanisms of action, such as oxidative stress, biofilm inhibition, adhesive inhibition, and toxic killing have been proposed,^1,13,14^ specific molecular mechanisms are rarely identified. Understanding the antifouling mechanism of action is therefore crucial for defining screening biomarkers and guiding the rational design of new agents.

The establishment of macrofouling on hard substrates is closely related to the composition of the existing biofilms of Gram-negative heterotrophic bacteria. Each macrofouling species is strongly and positively correlated with a specific biofilm bacterial composition, whereas other factors, such as substrate conditions, also affect the biofilm community structure.^15^ *P. tunicata* is a model species used in marine biofouling and biointerface studies.^7^ Lipopolysaccharide (LPS) present in the outer membrane of Gram-negative bacteria has been implicated in the formation and maintenance of Gram-negative bacterial biofilms, by promoting adherence to the surfaces and as a component of outer membrane vesicles essential for extracellular matrix communication and molecular trafficking, highlighting the importance of this component.^16,17^

Langmuir lipid monolayer models are a versatile tool used to study the interactions of small molecules with lipids at the molecular level. These models mimic the interface of a biomembrane and have been used to probe small molecules’ interaction with bacterial membranes.^18^ This work focused on the fundamental and applied research at the intersection of GBA26 with bacteria’s inner and outer membranes by using Langmuir lipid monolayer models, with different lipid compositions including LPS extracted from *P. tunicata*, and also by evaluating the membrane integrity in bacterial cells with fluorescent probes.

## 2 Materials and Methods

### 2.1 General

Phospholipids 1,2-dipalmitoyl-*sn*-glycero-3-phosphocholine (16:0, DPPC) (850355), 1,2-dipalmitoyl-*sn*-glycero-3-phosphoethanolamine (16:0, DPPE) (850705), and 1,2-dipalmitoyl-*sn*-glycero-3-phospho-(1′-rac-glycerol) (sodium salt) (16:0, DPPG) (840455) were obtained from Avanti Polar Lipids, Inc. (Alabaster, USA). *N*-(2-Aminoethyl)-3,4,5-trihydroxybenzamide hydrochloride (GBA26) was obtained *in house* from our library of polyphenolic compounds.^5^ LPS from *Pseudomonas aeruginosa* 10 (L9143) was obtained from Sigma-Aldrich. All lipids were used without further purification. Chemicals and solvents of analytical grade used were purchased from Sigma-Aldrich (Portugal). All solutions were prepared using ultrapure double-deionized water (conductivity inferior to 0.1 μS·cm^–1^). Vaatanen nine salt solution (VNSS) medium was prepared accordingly.^19^ UV–vis spectra were recorded on a Jasco V-730 spectrophotometer.

### 2.2 Bacteria Growth Conditions

*P. tunicata* (DSM 14096; DSMZ, Braunschweig, Germany) was used in this study to investigate the interactions between GBA26 and bacterial membranes, as well as its effects on metabolic activity, reactive oxygen species (ROS) production, and cell culturability. Before the experiments, bacteria were cultured overnight at 25 °C in VNSS marine medium supplemented with 15 g·L^–1^ agar (VWR International S.A.A., Fontenay-sous-Bois, France), prepared as previously described.^19^ Single colonies of *P. tunicata* were then inoculated into VNSS broth and incubated for 16 ± 2 h at 25 °C, 120 rpm. The bacterial suspension was centrifuged at 3772*g* (Eppendorf Centrifuge 5810R, Eppendorf, Hamburg, Germany), for 10 min at room temperature. The bacterial pellet was resuspended in fresh VNSS medium to achieve a final cell suspension with an optical density at 610 nm of 0.10, which corresponds to ∼1.0 × 10^8^ cells·mL^–1^.

### 2.3 *P. tunicata* Lipopolysaccharide Extraction

LPS from *P. tunicata* was extracted by following a hot phenol-water extraction protocol adapted from previous reports.^20,21^ Briefly, after growing *P. tunicata* in VNSS medium (see section 2.2.), the culture was centrifuged, and 6.6 g of biomass was obtained. Cells were washed by resuspending the pellet in 10 mM PBS (pH 7.4) and centrifuged at 10,000*g*, 5 min, 4 °C in ultracentrifuge Frontier FC5816R (Ohaus, New Jersey, USA). Cells were suspended in 10 mL of 20 mM Tris.HCl buffer (pH 7.2) and lysed under ultrasonication (pulse on/off 10 s/10 s, 15 min, 4 °C) on ice. Lysate was submitted to enzymatic digestion to eliminate nucleotide and protein contaminants by incubating with RNase A (100 μg·mL^–1^) and DNase I (50 μg·mL^–1^) along with 10 mM MgSO_4_, 1 mM CaCl_2,_ and 10 mM NaCl, at 37 °C, overnight, in an orbital shaker at 50 rpm. Subsequently, Proteinase K (100 μg·mL^–1^) and 0.5% SDS were added, and the mixture was incubated at 60 °C, 4 h, at 50 rpm. Liquified phenol at 90% (w/w) was prepared in a glass vial by weighing 9 g phenol crystals, adding 1 mL of purified water, and heating the solution at 68 °C under stirring in a water bath. The enzyme-digested lysate was equilibrated at 68 °C, then the 90% liquified phenol was added and heated at 68 °C for 30 min, 500 rpm. The mixture was cooled to 15 °C, transferred to a polypropylene vial, and centrifuged at 10,000*g*, 10 min, 15 °C. The water phase was collected into a new tube, and the phenol phase was re-extracted with 10 mL of purified water. Water phases were pooled, and 5 M NaCl and 3 M sodium acetate were added to adjust ionic strength (0.5 M) and pH (5). Afterward, 5 volumes of ethanol were added to precipitate LPS at −20 °C, overnight. The ethanolic mixture was centrifuged at 4000*g*, 4 °C, 15 min, and the supernatant was discarded. LPS pellet was reprecipitated by resuspending it in 5 mL of ultrapure water, with ionic strength and pH adjusted, and incubation at −20 °C, overnight, followed by centrifugation. The supernatant was discarded, and the LPS pellet was air-dried. The solid was suspended in 3 mL of ultrapure water, dialyzed with a MWCO of 1 kDa against 2.5 L of ultrapure water for 72 h, and lyophilized. In the end, 20 mg of *P. tunicata* LPS was obtained.

### 2.4 LPS Purity Analysis

Contaminant analysis of the LPS was executed by a UV spectrometer, evaluating the amount of polysaccharides, protein, and nucleotides by their spectroscopic absorbance at 204, 240, and 260 nm, respectively. The quality index of LPS (QI) was calculated according to eq 1, as described elsewhere.^20^

QI=(a×A204){(a×A204)+(b×A260)+(c×A280)}a(cofactor of lipids)=7.62b(cofactor of nucleic acids)=4.76c(cofactor of proteins)=102.05 1

LPS profiles from *P. tunicata* (PT) and *P. aeruginosa* (PA) were evaluated by electrophoresis and silver nitrate staining, as described elsewhere.^22^ Briefly, LPS samples were prepared in ultrapure water and sample buffer (50 mM Tris.HCl, pH 6.8, 10% glycerol, 2% SDS, 0.01% bromophenol blue, and 55 mM β-mercaptoethanol) and heated at 100 °C, 5 min. Samples were loaded (2 μg of each sample per well) in a SDS-polyacrylamide gel (4% stacking/12% resolving) and run in Tris-glycine-SDS buffer with 10 mA in the stacking gel and 20 mA in the separating gel. For the silver staining, all solutions were prepared immediately before use. The gel was photographed using a Bio-Rad ChemiDoc image apparatus.

### 2.5 Maldi-TOF Analysis

LPS from PA and PT fractions were analyzed using a MALDI-TOF Autoflex III instrument (Bruker GmbH, Bremen, Germany). The samples were suspended in 50% acetonitrile (ACN) containing 0.1% trifluoroacetic acid (TFA), at 10 mg·mL^–1^ and mixed in a ratio of 1:1 with a matrix of 2,5-dihydroxybenzoic acid (10 μg·mL^–1^ 50% ACN/0.1% TFA). The MALDI-TOF MS spectra were obtained in negative-ion reflectron mode (500–3500 *m*/*z*) and positive-ion linear mode (1000–20,000 *m*/*z*). The acquired mass spectra were calibrated and processed under computer control by using the Bruker Daltonics flexAnalysis software.

### 2.6 Langmuir Surface Pressure–Area Isotherms

DPPC was solubilized in chloroform (1 mg·mL^–1^), and DPPE and DPPG in chloroform:methanol (6:4) (1 mg·mL^–1^). LPS extracts from PA and PT were suspended in chloroform:methanol (8:2) (0.5 mg·mL^–1^) using 6 cycles of 30 min in ultrasounds. GBA26 was dissolved in the subphase (10 mM HEPES buffer, pH 7.4, and 100 mM NaCl) at 50 μM. Surface pressure–area isotherm experiments were performed as previously described.^23^ Briefly, lipids were spread onto a subphase in a KSV NIMA Langmuir trough of 420 mL at 21 ± 1 °C, with or without compound. Thereafter, monolayers were allowed to equilibrate for 10 min (phospholipids) or 20 min (LPS) before compression to ensure interfacial stabilization. Surface pressure –area isotherms were then obtained through the compression made by two symmetric barriers at a rate of 5 Å^2^/molecule/min, and the surface tension was measured using a Wilhelmy microbalance, with a filter paper plate with an accuracy superior to 0.1 mg·mL^–1^. Before each experiment, the trough was cleaned with ethanol and ultrapure water. Each experiment was performed in triplicate and all replicates were plotted in Figure S1, Supporting Information. Analysis was performed with Origin software 8.0 (OriginLab Corporation, Northampton, MA, USA), graphs were constructed with GraphPad Prism version 7.0 (GraphPad Software, Boston, MA, USA), and data is presented as the mean ± standard deviation. Isotherms are presented as surface pressure (π, mN·m^–1^) dependent on the area per molecule (*A*, Å^2^). The minimum molecular area (A_0_) was determined from the compression isotherms by extrapolating the tangent to the steepest linear region of the curve at high surface pressures, just before collapse (typically between 50 and 55 mN·m^–1^), to zero surface pressure. In this region, the isotherm was approximated by a linear relation (eq 2), and A_0_ was obtained from the intercept at zero surface pressure (eq 3). This procedure provides an estimate of the limiting molecular area in the condensed phase. Similarly, other lipid areas, namely A_10_ and A_30_, were calculated by extrapolating the tangent at specific surface pressures (10 and 30 mN·m^–1^), respectively.^24^

$$\begin{array}{l} \pi =mA+b\\ A=\mathrm{Area per molecule}\;\left({Å}^{2}\right)\\ \pi =\mathrm{Surface pressure}\;\left(mN\text{·}m^{-1}\right) \end{array}$$ 2

3 $$A_{0}=-\frac{b}{m}$$

The compressibility modulus (C_s_^–1^), which reflects the elastic resistance of the monolayer to lateral compression, is defined by the differential expression represented in eq 4.δ∂

$$\begin{array}{l} C_{s}^{-1}=-A{\left(\frac{\partial \pi }{\partial A}\right)}_{T} \end{array}$$ 4

In practice, C_s_^–1^ was calculated from the experimental compression isotherms using a finite-difference approximation of this differential relation.^25^ The maximum compressibility modulus (C_s_^–1^_max_) was determined after smoothing the curve with the adjacent-averaging method (5 points).

For Langmuir monolayers, the ideal additivity rule assumes that, in the absence of specific interactions (no attraction or repulsion), each component (lipid), in a mixed monolayer, occupies the same molecular area as in its corresponding pure monolayer at a given surface pressure. Any deviation from this ideal behavior is captured by the excess area per molecule, A_exc_. Thus, for binary mixtures, the ideal mean molecular area (*A_ideal_*) at a given surface pressure (π) was calculated as in eq 5 and for ternary mixtures as in eq 6:^26^

5 $$A_{ideal}\left(\pi \right)=x_{1}A_{1}\left(\pi \right)+x_{2}A_{2}\left(\pi \right)$$

6 $$A_{ideal}\left(\pi \right)=x_{1}A_{1}\left(\pi \right)+x_{2}A_{2}\left(\pi \right)+x_{3}A_{3}\left(\pi \right)$$

where A_1_, A_2_, and A_3_ are the extrapolated mean molecular areas of the pure component in the single monolayers at the same surface pressure, and x_1_, x_2_, and x_3_ are their respective molar fractions in the mixed monolayers (namely DPPC, DPPE and DPPG). The excess area per molecule (A_exc_) was then calculated as in eq 7.^26^

$$A_{exc}\left(\pi \right)=A_{mix}‐A_{ideal}$$ 7

where A_mix_ corresponds to the experimentally measured mean molecular area of the binary or ternary monolayer. Positive and negative values of A_exc_ indicate repulsive and attractive interactions between components, respectively.

### 2.7 Brewster Angle Microscopy

Microscopic images of the lipid domains were obtained by a KSV NIMA Brewster Angle Microscope (BAM), with a lateral resolution of 2 μm, coupled to a Langmuir trough for real-time surface pressure readings. Conditions similar to those used in the measurements of the surface pressure–area isotherms were used, and all images were recorded at 21 ± 1 °C. For better contrast, the images were treated by adjusting the brightness and removing the background. Pixel greyscale histograms were analyzed with ImageJ^65^ and plotted using GraphPad Prism version 7.0 (GraphPad Software, Boston, MA, USA), as described elsewhere.^27^

### 2.8 Antioxidant Property Evaluation

#### 2.8.1 2,2-Diphenyl-1-picrylhydrazyl (DPPH) Assay

Briefly, 2.5–20 μL of 1 mg·mL^–1^ GBA26 or gallic acid solutions in DMSO were added to 2 mL of a freshly prepared 200 μM solution of DPPH in ethanol, and the mixtures were kept under vigorous stirring at room temperature.^28,29^ After 10 min, the absorbance at 515 nm was measured. (±)-6-hydroxy-2,5,7,8-tetramethylchromane-2-carboxylic acid (Trolox) (0.5 mg·mL^–1^) was used as a reference antioxidant. Data were expressed as EC_50_ values and experiments were run in triplicate.

#### 2.8.2 Ferric Reducing/Antioxidant Power (FRAP) Assay

Briefly, 5–30 μL of 1 mg·mL^–1^ GBA26 or gallic acid solutions in DMSO were added to 3.6 mL of a 1.7 mM FeCl_3_ and 0.83 mM 2,4,6-tris­(2-pyridyl)-s-triazine (TPTZ) solution in 0.3 M acetate buffer (pH 3.6).^30^ The mixture was taken under vigorous stirring at room temperature, and after 10 min, the absorbance at 593 nm was measured. Results were expressed as Trolox equivalents, and experiments were run in triplicate.

### 2.9 Characterization of the Antibacterial Effects of GBA26 on *P. tunicata*

The antibacterial mechanisms of GBA26 against *P. tunicata* were characterized through flow cytometry. Bacteria were exposed to GBA26 at a concentration of 12.28 μg·mL^–1^ at 25 °C, for 24 h. Subsequently, cells were stained in the absence of light for 30 min with bis­(1,3-dibutylbarbituric acid) trimethine oxonol (DiBAC_4_(3); Sigma-Aldrich, Taufkirchen, Germany) at 2.5 μg·mL^–1^ to evaluate cell membrane potential, propidium iodide (PI, Invitrogen Life Technologies, Alfagene, Lisboa, Portugal) at 2.5 μg·mL^–1^ to assess cell membrane integrity, 5(6)-carboxyfluorescein diacetate (CFDA; Sigma-Aldrich, Taufkirchen, Germany) at 5 μg·mL^–1^ to measure cell metabolic activity, and 2′,7′-dichlorofluorescein diacetate (DCFH-DA, Sigma-Aldrich, Taufkirchen, Germany) at 25 μM to detect the endogenous reactive oxygen species (ROS) production. Three independent assays were performed. The effect of GBA26 on these bacterial parameters was investigated by acquiring 20,000 cells at a flow rate of 30 μL·min^–1^ in a CytoFLEX flow cytometer model V0–B3-R1 (Beckman Coulter, Brea, CA, USA). The results were analyzed using CytExpert software (version 2.4.0.28, Beckman Coulter, Brea, CA, USA) and presented as the mean intensity of fluorescence (MIF) at 525/40 nm for DiBAC_4_(3), CFDA, and DCFH-DA, and at 585/42 nm for PI. In parallel, the effect of GBA26 on *P. tunicata* culturability was evaluated by exposing the bacteria to the conditions described above, plating the bacterial suspension on VNSS agar, and enumerating colony forming units (CFUs). The results are presented as the mean and standard deviation from three independent assays.

### 2.10 Statistical Analysis

Descriptive statistics were used to calculate the mean and standard deviation for the fluorescence intensity of bacterial cells analyzed through flow cytometry and the number of culturable cells. A *t*-test for independent samples was used to evaluate differences in cell fluorescence intensity between each treatment condition, while differences in cell culturability were evaluated using the Mann–Whitney test. Statistically significant differences were considered for *p*-values < 0.05. Data analysis was performed using the IBM SPSS Statistics version 27.0 for Windows (IBM SPSS, Inc., Chicago, IL, USA).

## 3 Results and Discussion

### 3.1 Interaction of GBA26 with Monolayer Membrane Models

The antifouling activity of GBA26 (Figure 1A) was observed in solution and when incorporated in a coating matrix, immobilized or not,^5,7^ raising the possibility that GBA26 action could occur upon the contact of the organism with the coated surface. Investigating the possible interactions between GBA26 and biological membranes could open avenues to understand the mechanism of action of GBA26 antibiofilm activity against *P. tunicata*. To this aim, different lipid monolayer models mimicking the inner and outer membranes of bacteria were prepared, and the changes in surface pressure in dependence on the average area occupied per lipid molecule were analyzed using a Langmuir trough in the absence and presence of GBA26 in the subphase. To obtain surface pressure–area isotherms, the water-insoluble lipids were spread on an aqueous subphase and, upon compression, the surface area was reduced, forcing the molecules to reorganize at the interface, meaning that the monolayer undergoes different phase transitions (gaseous, liquid-expanded, liquid-condensed, and condensed). The reorganization depends on the lipid structure (charge and size of the polar head and the saturation of the hydrophobic tails), solvation, and the presence or absence of molecules that interact with the lipids.^31,32^

#### 3.1.1 Inner Membrane

Gram-negative bacteria’s inner membranes are constituted mainly by phosphatidylethanolamine (DPPE, Figure 1A), phosphatidylglycerol (DPPG, Figure 1A), and cardiolipin (75:20:5).^33^ Additionally, the acyl chains in these bacterial membranes are generally shorter and more saturated.^34^ Particularly, different strains of *Pseudoalteromonas* have been reported to have their inner membrane mainly constituted by DPPE and DPPG in a proportion of 75:25.^33^ Ciumac and colleagues reported a binary mixed lipid monolayer model of the bacterial membrane by using a mix of DPPE:DPPG in 7:3 ratio to study the effect of antimicrobial peptides.^35^

We began by using a phosphatidylcholine (DPPC, Figure 1A) monolayer as the initial model and then constructed a bacterial inner membrane model based on DPPC to achieve well-defined transition states. The model was developed stepwise to assess the influence of different phospholipid polar heads (DPPC, DPPE, DPPG) on monolayer behavior, both in the absence and presence of GBA26, using single, binary, and ternary systems. HEPES-buffered saline at pH 7.4 was used as subphase to mimic the bacterial intracellular pH (7.2 to 7.8),^36^ and GBA26 was dissolved in the buffer.

**Table 1:** Area per lipid molecule and the maximum compressibility modulus of phospholipid monolayers, in the absence and presence of GBA26 (50 μM)^a^

Monolayer	Chemical	C_s_^–1^_max_ (mN·m^–1^)	A_0_ (Å^2^)	A_10_ (Å^2^)	A_30_ (Å^2^)
DPPC	–	200 ± 3	41.8 ± 0.5	53.2 ± 1.9	42.6 ± 0.4
	GBA26	142 ± 16	36.5 ± 1.0	91.1 ± 2.7	36.7 ± 0.7
					
DPPC:DPPE(7:3)	–	256 ± 23	45.2 ± 1.3	52.0 ± 1.2	45.9 ± 0.9
	GBA26	185 ± 21	43.8 ± 1.4	73.6 ± 5.2	48.3 ± 1.1
					
DPPC:DPPG(9:1)	–	200 ± 4	42.2 ± 0.2	49.9 ± 0.8	42.4 ± 0.2
	GBA26	171 ± 5	38.3 ± 0.8	76.6 ± 6.1	40.2 ± 1.1
					
DPPC:DPPE:DPPG(6:3:1)	–	253 ± 20	51.7 ± 1.4	57.7 ± 1.8	51.9 ± 0.8
	GBA26	217 ± 5	52.3 ± 0.7	68.8 ± 1.3	54.0 ± 0.7

a C_s_^–1^_max_: maximum compressibility modulus; A_0_: minimum area per lipid molecule; A_10_: area per lipid molecule at 10 mN·m^–1^ surface pressure; A_30_: area per lipid molecule at 30 mN·m^–1^ surface pressure.

Regarding the profile of the monolayers without GBA26, it was observed that the obtained isotherms’ profiles (Figure 1B) corresponding to the pure DPPC, DPPE, DPPG, and mixed monolayers were in agreement with those already reported.^37–39^ The monolayer corresponding to the pure DPPC showed a transition from the gaseous phase to the liquid-expanded phase at 100 Å^2^, a liquid-expanded to liquid-condensed plateau at a surface pressure <10 mN·m^–1^, and a condensed phase from >15 mN·m^–1^ until the monolayer collapses at a surface pressure of approximately 55 mN·m^–1^ with a minimum area per molecule (A_0_) of 41.8 ± 0.5 Å^2^ and maximum compressibility modulus (C_s_^–1^_max_) of 200 ± 3 mN·m^–1^ (Table 1). Pure DPPE monolayer exhibited a liquid-expanded to liquid-condensed phase transition without defined lipid phases or plateaus, until the monolayer collapses at a surface pressure of approximately 43 mN·m^–1^ with a minimum area per molecule (A_0_) of 73.8 ± 0.8 Å^2^ and maximum compressibility modulus (C_s_^–1^_max_) of 92 ± 3 mN·m^–1^ (Table S1, Supporting Information). Pure DPPG monolayer presented a transition from the gaseous phase to the liquid-expanded phase at 120 Å^2^, a liquid-expanded to liquid-condensed plateau at a surface pressure at ∼10 mN·m^–1^, and a condensed phase from >15 mN·m^–1^ until the monolayer collapses at a surface pressure of approximately 65 mN·m^–1^ with a minimum area per molecule (A_0_) of 43.7 ± 0.2 Å^2^ and maximum compressibility modulus (C_s_^–1^_max_) of 187 ± 4 mN·m^–1^ (Table S1, Supporting Information). The presence of 10% of DPPG did not significantly alter the isotherm profile of DPPC; only a slight decrease of the plateau of the liquid-expanded to liquid-condensed phase transition was observed (Figure 1B), as DPPG’s negatively charged polar head alters slightly its cylindrical geometry due to electrostatic repulsion,^40^ disturbing the highly cooperative phase transition characteristic of DPPC as observed previously.^41^ However, in the presence of 30% of DPPE, in the binary system DPPE:DPPE (7:3), a suppression of the liquid-expanded to liquid-condensed phase transition was observed, as well as in the ternary system (DPPC:DPPG:DPPE (6:3:1)), where the effect of DPPE prevailed (Figure 1B).^35^ When DPPE was present in the monolayer, an increase in the minimum area per molecule occured in the condensed phase when compared to pure DPPC (Table 1). Such an effect was more pronounced in the ternary system, probably due to the presence of DPPG in the mixture, highlighting the role of the charges and geometry of DPPG and DPPE polar heads and their solvation. The increase of the area per molecule was also reported in the binary mixture of DPPE:DPPE (7:3), when comparing with the area per molecule of the pure lipids.^35^ Concomitantly, the maximum compressibility modulus (C_s_^–1^_max_) increased in the presence of DPPE (Table 1), indicating a less compressible and more rigid monolayer when compared to pure DPPC and DPPC:DPPG (9:1) monolayers. Higher compressibility modulus values reflect increased elastic resistance to lateral compression, consistent with the dense packing promoted by DPPE’s smaller ethanolamine headgroup and inverted cone-like geometry, which favor nonplanar lipid aggregates.^35,42^ Accordingly, DPPE-containing mixed monolayers displayed reduced lateral fluidity, as evidenced by the more condensed isotherms obtained (Figure 1B). Further, the effects of the DPPE and DPPG on mixed monolayers were assessed by excess area per molecule (A_exc_) analysis, which provides insight into the nature of intermolecular interactions within the mixed monolayers. A_exc_ close to zero indicate near-ideal mixing behavior (miscibility of the components), whereas negative and positive values reflect attractive and repulsive interactions between unlike components, respectively.^26^ For DPPC:DPPE (7:3) monolayers, A_exc_ values were negative at all investigated surface pressures (Table S2, Supporting Information), indicating attractive interactions and enhanced packing efficiency. This behavior is consistent with favorable hydrogen bonding interactions between the DPPC and DPPE headgroups, leading to tighter molecular packing. In contrast, the DPPG-containing monolayers, DPPC:DPPG (7:3) and DPPC:DPPE:DPPG (6:3:1) mixtures, exhibited A_exc_ values close to zero at A_0_ and A_30_, although slightly positive. These observations suggest ideal miscibility or weak repulsive interactions, which can be attributed to headgroups mismatch and steric constraints under highly compressed conditions in the condensed phase. Notably, at A_10_, negative A_exc_ values were observed for all studied mixtures, reflecting enhanced attractive interactions during the liquid-expanded to liquid-condensed phase transition. These results highlight the competing effects of electrostatic interactions and molecular packing in determining the lateral organization of multicomponent lipid films.

**Figure 2:**
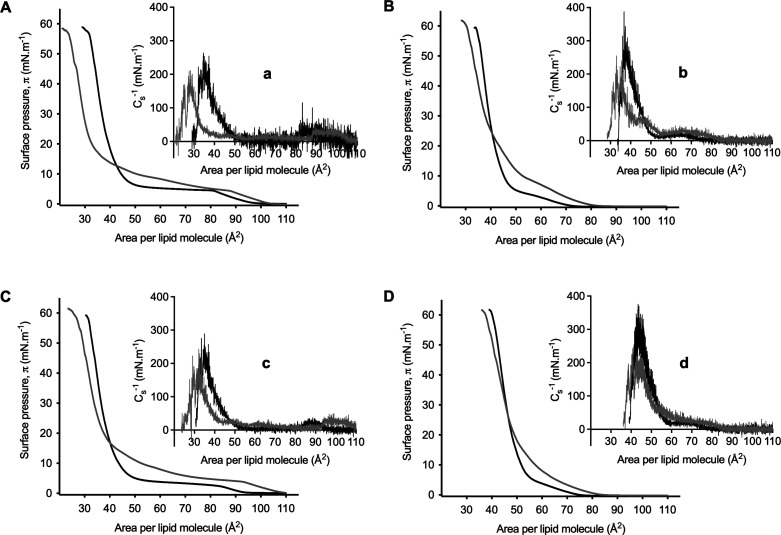
GBA26 interaction with phospholipid monolayers. Surface pressure–area isotherms of monolayers consisting of (A) DPPC, (B) DPPC:DPPE (7:3), (C) DPPC:DPPG (9:1), and (D) DPPC:DPPE:DPPG (6:3:1) measured in 10 mM HEPES, 100 mM NaCl (pH 7.4) at 21 °C, in the absence (black) and presence (gray) of GBA26 (50 μM). Insets a–d are the compressibility modulus (C_s_^–1^) graphs corresponding to the isotherms represented. Only one representative experiment of three replicates is presented per each condition.

GBA26 showed to interact with the DPPC monolayer, changing both the area per molecule and the compressibility moduli when compared to DPPC monolayer without GBA26 (Figure 2A; Table 1). In the liquid-expanded phase, GBA26 caused a marked expansion of the monolayer, as evidenced by the increase in the area per molecule at 10 mN·m^–1^ (A_10_ increased from 53.2 ± 1.9 to 91.1 ± 2.7 Å^2^, Table 1). In this region, a slight increase in the compressibility moduli is observed (Figure 2A, inset a), indicating a more rigid monolayer organization despite the expanded state. Conversely, in the condensed phase, GBA26 caused a decrease in the minimum area per molecule (A_0_ decreased from 41.8 ± 0.5 to 36.5 ± 1.0 Å^2^, Table 1), suggesting a tighter lipid packing. However, this effect was accompanied by pronounced decrease in the maximum compressibility modulus (C_s_^–1^_max_ from 200 ± 3 to 142 ± 16 mN·m^–1^, Table 1), indicating a more compressible monolayer and less rigid monolayer in the presence of GBA26. Interestingly, this phenomenon was previously reported for peptides and arginine-based surfactants attributed to lipid solubilization or packing disruption effects.^41,43^ GBA26 also interacted with the lipid mixed monolayers and a similar qualitative behavior was observed for these monolayers in the presence of GBA26, with increased molecular areas and higher compressibility moduli in the liquid-expanded phase, and reduced molecular areas together with lower compressibility moduli in the condensed phase (Figure 2B–D, insets b–d). Notably, the magnitude of these effects was generally reduced in mixed systems compared to pure DPPC monolayer. In DPPC:DPPG (9:1) and DPPC:DPPE (7:3) monolayers, GBA26 increased A_10_ by approximately 27 and 21 Å^2^, respectively, whereas an increase of approximately 38 Å^2^ was observed for pure DPPC (Table 1). For the ternary DPPC:DPPE:DPPG (6:3:1) system, the expansion of the liquid-expanded phase is further attenuated, with an increase of approximately 11 Å^2^. These lower increments of the liquid-expanded phase in the mixed monolayers *vs* the pure DPPC monolayer in the presence of GBA26 could be attributable to the attractive forces among the different lipidic components, as discussed above (Table S2, Supporting Information). Likewise, in the condensed phase, although GBA26 decreased the maximum compressibility modulus in all tested systems, the reduction was substantially larger for pure DPPC (∼58 mN·m^–1^) and DPPC:DPPE (7:3) (∼71 mN·m^–1^) monolayers than for the DPPG-containing monolayers (∼30 mN·m^–1^; Table 1). In the ternary system, the minimum molecular area (A_0_) remained essentially unchanged upon GBA26 addition (Table 1). Although GBA26 induced only modest changes in the minimum area per molecule, it produced a comparatively larger reduction in C_s_^1–^_max_ in DPPC:DPPE (7:3) than in pure DPPC monolayers, relative to their respective baseline rigidities, indicating that the presence of DPPE amplified the GBA26-induced fluidization of the condensed phase. This suggests that GBA26 primarily perturbs lateral cohesion and elastic resistance (e.g., headgroup interaction networks and/or condensed-phase organization) rather than simply shifting the limiting packing area. Overall, these results underscore the role of lipid headgroup chemistry and geometry in modulating the extent of GBA26-induced membrane perturbation.

The monolayer morphology was also analyzed by using Brewster angle microscopy (BAM). BAM applied to monolayers’ study is based on the changes in the reflectivity of the polarized laser beam when it reaches a condensed monolayer, allowing the visualization of the shape and texture of the lipid domains as surface pressure increases.^44^ Comparing the different monolayers of DPPC and mixtures with DPPE or/and DPPG, differences in the shape and size of the condensed phase domains can be observed (Figure 3). DPPC alone showed condensed phase domains with characteristic clover-like shapes^45,46^ that start to form at a surface pressure higher than 5 mN·m^–1^. At 10 mN·m^–1^, it was possible to visualize grown lipid domains that start to clash to render the condensed phase observed at 20 and 30 mN·m^–1^. When the monolayer had 10% DPPG, no evident differences were detected regarding the size and shape of the lipid domains and the surface pressures to reach a generalized condensed state. However, mixtures with 30% DPPE started to form condensed phase domains at lower surface pressures, being visible at 5 mN·m^–1^, and the domains were smaller and presented a circular shape. In general, upon GBA26 presence in the subphase, the formation and evolution of the lipid domains were affected, with overall lower condensation level for surface pressures lower than 30 mN·m^–1^ in all tested monolayers, as depicted in the condensation level graphs (Figure 3). Interestingly, the condensation levels also revealed that the effect of GBA26 was stronger in a 100% DPPC monolayer than in the mixed monolayers, corroborating the surface pressure–area isotherms analysis, when comparing the areas per molecule at the same surface pressures of the different isotherms. Moreover, a 100% DPPC monolayer in contact with GBA26, at 30 mN·m^–1^, showed a lower condensation level than the DPPC alone. BAM images also showed that GBA26 increased the size of the lipid domains of the mixed monolayer of DPPC:DPPE:DPPG (6:3:1), indicating that interactions among the compound and the delicate system of lipids and solvation layer may occur, affecting the overall lipid organization in the monolayer. Such interaction effects were reviewed elsewhere and classified as alteration of membrane physical curvature, lipid clustering, packing defects with complete or partial loss of permeability, and direct targeting of certain lipids.^47^

**Figure 3:**
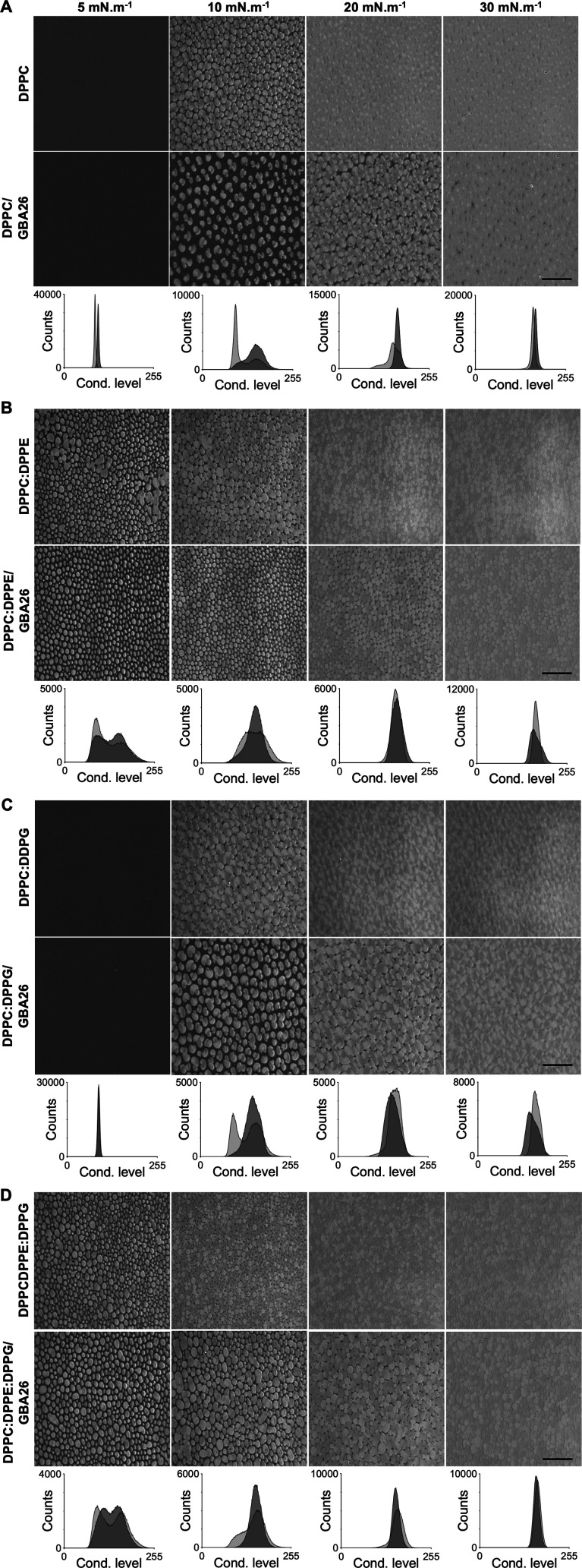
The effects of GBA26 on the lipid domain formation and condensation levelsof the monolayer. Brewster angle microscopic images (BAM) of Langmuir monolayers of (A) DPPC, (B) DPPC:DPPE (7:3), (C) DPPC:DPPG (9:1), and (D) DPPE:DPPE:DPPG (6:3:1) at 5, 10, 20, 30 mN·m^–1^ surface pressures, in 10 mM HEPES, 100 mM NaCl (pH 7.4) at 21 °C. The top and bottom rows of each set correspond to the monolayer on the subphase without and with GBA26 at 50 μM, respectively. Scale bar represents 100 μm. Graphs represent the condensation level histograms based on the pixel grayscale of the monolayers' BAM images in the absence (dark gray) and presence (light gray) of GBA26. Cond. level: condensation level.

Overall, these results demonstrate for the first time that GBA26 interacts with lipids in a monolayer, indicating that this antifouling compound has the potential to induce alterations in biological membranes, particularly bacteria’s inner membrane.

#### 3.1.2 Outer Membrane

LPS has been the subject of Langmuir monolayer studies to understand the importance of the LPS structure and the interaction of different classes of antibiotics.^48^ Therefore, this study aimed to understand if the antibiofilm compound GBA26 could interact with LPS. For that, two LPS monolayer models were established to mimic the bacteria’s outer membrane, similarly to reported works.^18^ Commercially available LPS extract from the terrestrial bacterium *P. aeruginosa* was acquired, and LPS from the marine bacterium *P. tunicata* was extracted. The extraction of the LPS from *P. tunicata* was performed using the hot phenol-water procedure, with previous enzymatic digestion and subsequent dialysis. Enzymatic digestion with DNase I, RNase A and protein K showed to be efficient in diminishing nucleic acids and removing proteins, as demonstrated by the lower absorbances at 260 and 280 nm, respectively, when comparing to LPS from *P. aeruginosa* (extracted by trichloroacetic acid method according to the manufacturer) (Figure 4A). LPS from *P. tunicata* was successfully obtained (20 mg) with a yield of 0.3% toward wet cells weight, with a calculated quality index (eq 1, Section 2.4) of 68%, comparable to the quality index of the commercial LPS from *P. aeruginosa*, about 53%, when applying the same method.^20^ SDS-page analysis (Figure 4B) indicated that the extracted LPS from the marine bacterium *P. tunicata* was constituted by two well-defined bands, one with 80 kDa and another lower than 6 kDa, referring to smooth (lipid A, core, and O-antigen) and rough (lipid A and core) LPS types, respectively. Interestingly, *P. tunicata* LPS did not exhibit a ladder-like profile, contrasting with the profile of *P. aeruginosa* LPS (Figure 4B) and other bacteria.^49^ Indeed, previous reports on LPS from *Pseudoalteromonas* sp. also demonstrated this lack of multiple LPS O-antigen polysaccharide lengths.^50,51^ Mass spectrometry based on MALDI-TOF analysis allowed to determine the molecular weight of the most abundant forms of LPS (polysaccharide chain + lipid A) and lipid A (the lipidic portion that is inserted in the outer membrane) of the commercial LPS from *P. aeruginosa*(*LPS*_[*M*+*H*]_^+^ = 5800 *m*/*z* and *lipid A*_[*M*‑*H*]_^–^ = 1302 *m*/*z*), as well as of the extracted LPS from *P. tunicata*(*LPS*_[*M*+*H*]_^+^ = 3450 *m*/*z* and *lipid A*_[*M*‑*H*]_^–^ = 1235 *m*/*z*) (Figures S2 and S3, Supporting Information). The determined lipid A molecular weights are in accordance with the previously reported lipid A molecular weight ranges for related bacteria strains.^50,52^

**Figure 4:**
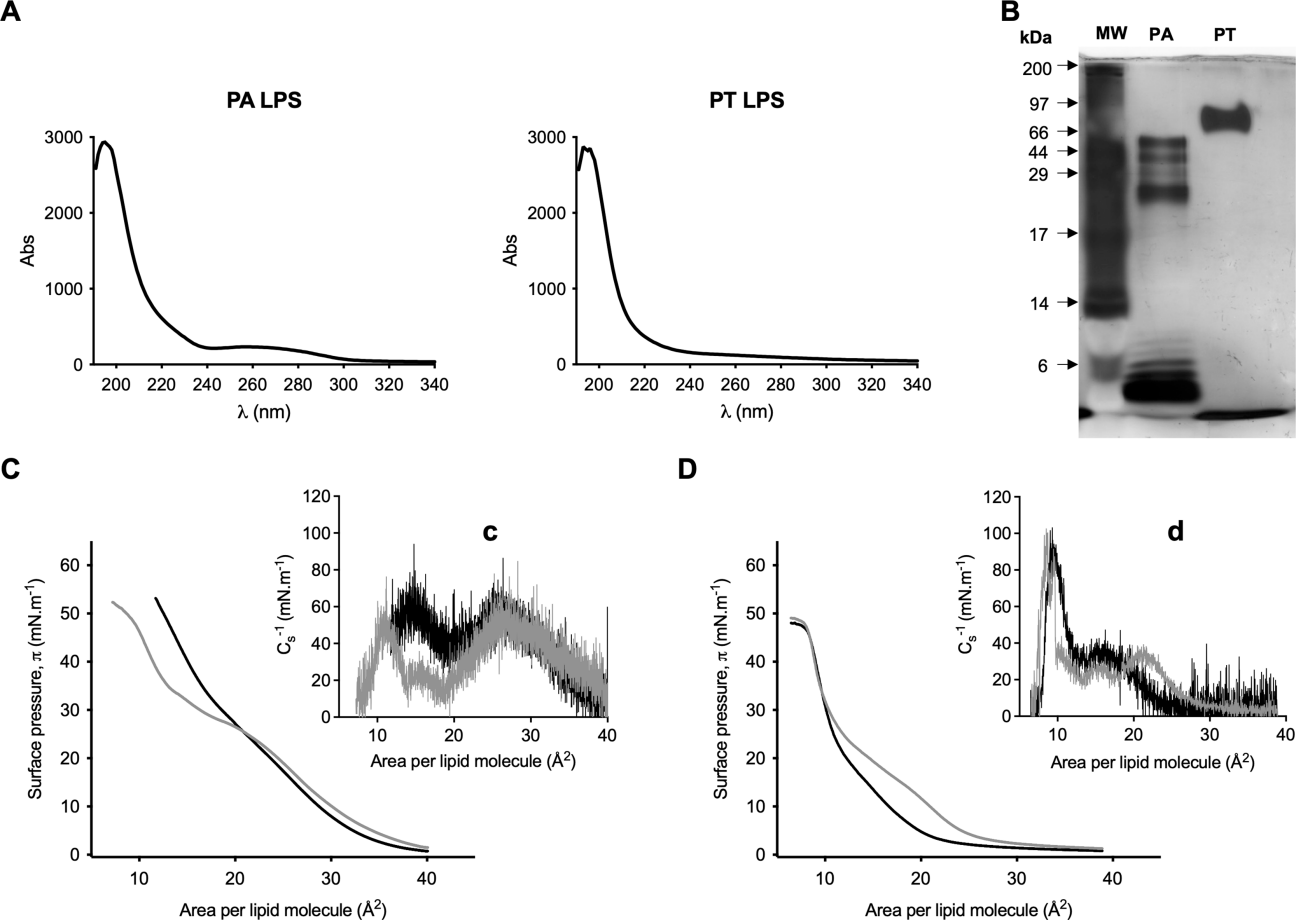
Quality characterization of LPS from *Pseudomonas aeruginosa* and *Pseudoalteromonas tunicata*, and interaction of GBA26 with LPS monolayers. (A) UV spectra (340–190 nm) of lipopolysaccharides from *P. aeruginosa* (PA LPS) and from *P. tunicata* (PT LPS). Lipopolysaccharides absorb at lower wavelengths with a maximum absorbance at approximately 200 nm, nucleic acids absorb at 260 nm, and proteins absorb at 280 nm. (B) The LPS profile was analyzed by SDS-page with silver staining. Molecular weight (MW) ladder (sc-2361). (C, D) Surface pressure–area isotherms of monolayers consisting of (C) 100% LPS from *P. aeruginosa* and (D) 100% extracted LPS from *P. tunicata* measured in 10 mM HEPES, 100 mM NaCl (pH 7.4), at 21 °C, in the absence (black) and presence (gray) of GBA26 (50 μM). Insets (c) and (d) are the compressibility modulus (C_s_^–1^) graphs corresponding to the isotherm represented. Only one representative experiment of three replicates is presented per each condition.

Thereafter, lipopolysaccharides were used in Langmuir lipid monolayer models by spreading them on HEPES-buffered saline (pH 7.4) as the subphase. *P. aeruginosa* LPS monolayer alone presented a surface pressure–area isotherm without lipid phase plateaus (Figure 4C), as reported previously.^53^ In the presence of GBA26, a slight increase of the area per molecule was observed for surface pressures lower than 25 mN·m^–1^ with lower compressibility moduli for the respective areas per molecule (around 20 to 25 Å^2^), which corresponds to more compressible monolayers, indicating that GBA26 induced fluidization of the monolayer (Figure 4C, inset c). At surface pressures of 25–35 mN·m^–1^, stronger discontinuities on the lipid phase were produced in the presence of GBA26 with a significant decrease in the compressibility moduli. However, at these surface pressures and higher, a significant decrease in the minimum area per molecule (A_0_ from 25.4 ± 0.7 to 20.7 ± 0.3 Å^2^, Table 2) was observed, suggesting that GBA26 contributes to a rearrangement of lipid packing at higher surface pressures.^53,54^

**Table 2:** Area per lipid molecule^a^ and maximum compressibility modulus of LPS monolayers, in absence and presence of GBA26 (50 μM)

Monolayer	Chemical	C_s_^–1^_max_ (mN·m^–1^)	A_0_ (Å^2^)	A_10_ (Å^2^)	A_30_ (Å^2^)
**LPS** *P. aeruginosa*	–	60 ± 3	25.4 ± 0.7	34.9 ± 0.5	31.9 ± 0.5
	GBA26	57 ± 2	20.7 ± 0.3	37.9 ± 0.9	37.5 ± 0.8
**LPS** *P. tunicata*	–	97 ± 8	13.1 ± 0.3	22.4 ± 1.9	14.3 ± 0.4
	GBA26	92 ± 6	13.2 ± 0.5	26.5 ± 0.1	16.1 ± 2.1

a The area per lipid molecule values of LPS are underestimated. They were calculated based on the determined lipid A molecular weights by MALDI-TOF, as a representative species of the extract. C_s_^–1^_max_: maximum compressibility modulus; A_0_: minimum area per lipid molecule; A_10_: area per lipid molecule at 10 mN·m^–1^ surface pressure; A_30_: area per lipid molecule at 30 mN·m^–1^ surface pressure.

The monolayer condensation was visualized with BAM and no condensed lipid domains were observed. Instead, a condensed monolayer, like a film, was immediately identified at lower surface pressures and behaved constantly throughout pressure application (Figure S4, Supporting Information).

After this result, we studied the interaction of GBA26 with LPS extracted from *P. tunicata*, the target species for GBA26 antibiofilm activity. *P. tunicata* LPS monolayer presented a distinctive isotherm from *P. aeruginosa* LPS, showing a liquid-expanded to liquid-condensed phase transition and a condensed phase (Figure 4D). On another note, LPS monolayer from *P. tunicata* demonstrated a higher maximum compressibility modulus compared to LPS monolayer from *P. aeruginosa* (C_s_^–1^_max PT_ = 97 ± 8 mN·m^–1^ and C_s_^–1^_max PA_ = 60 ± 3 mN·m^–1^, respectively, Table 2), indicating a less compressible and more rigid monolayer. Interestingly, LPS from *P. aeruginosa* also presented higher molecular weights, suggesting the presence of longer oligosaccharide chains. This observation aligns with previous findings that LPS with longer oligosaccharide chains tend to form more deformable monolayers with lower compressibility moduli,^55^ likely due to steric and electrostatic interactions mediated by the LPS core or O-antigen. These effects influence molecular packing and reinforce the link between chain length and increased monolayer fluidity.^53^

GBA26 showed to interact with *P. tunicata* LPS, increasing the area per molecule in the liquid-expanded to liquid-condensed phase transition (A_10_ from 22.4 ± 1.9 to 26.5 ± 0.1 Å^2^, Table 2, Figure 4D). This interaction induced a phase discontinuity in the LPS monolayer, accompanied by an increase in the compressibility moduli at those areas per molecule (Figure 4D, inset d), suggesting that GBA26 expanded the lipid molecular area while simultaneously rigidifying the monolayer. This effect contrasts with its interaction with LPS from *P. aeruginosa*, where a decrease in the compressibility moduli for the same areas per moleculewas observed , inducing monolayer fluidization (Figure 4C, inset c). Also, in opposition to *P. aeruginosa* LPS monolayer, the GBA26 did not elicit differences in the lipid packaging in the condensed phase of the *P. tunicata* LPS monolayer (similar A_0_ and A_30_ values in the absence and presence of GBA26, Table 2).

These results show the interaction of GBA26 with the *P. aeruginosa* and *P. tunicata* LPS, hypothesizing alterations in the bacterial outer membrane. Further analysis is fundamental to understanding if the interaction occurs at the O-antigen, core, or lipid A level of the LPS structure. Cetuk and colleagues argued that hydrophobic antibiotics were capable of intercalating the lipid A of LPS in the liquid-expanded phase and would be squeezed out upon compression, whereas cationic antibiotics would intercalate the polar layer and stably increase the areas per molecule. Such stable interaction, even at higher surface pressures, indicated the possible mechanism of self-permeation of this type of molecules by deformation of the LPS layer.^48^ According to the origin of the LPS, GBA26 demonstrated different effects on the compressibility of the LPS monolayers, suggesting that the constitution of LPS influences not only the surface pressure–area isotherm profile but also the type of interactions with GBA26. Overall, these results point out the putative antibiofilm mechanism of GBA26 by interacting with bacteria’s outer membrane. Moreover, the interfacial behavior of GBA26 observed here can be rationalized in terms of the polarity and predicted ionization state of GBA26 (both positively charged and zwitterionic species), which possibly govern its affinity for phospholipids and LPS interfaces through polar interactions.

### 3.2 Characterization of Effects of GBA26 on *P. tunicata*

To understand if the observed effect of GBA26 on monolayer membrane models correlates with the membranes of live bacteria, a membrane permeability and metabolic activity study was carried out. For this purpose, *P. tunicata* cells were exposed to 12.28 μg·mL^–1^ of GBA26 for 24 h (concentration used in the Langmuir monolayer study). Subsequently, cells were stained with bis­(1,3-dibutylbarbituric acid) trimethine oxonol (DiBAC_4_(3)), propidium iodide (PI), 5(6)-carboxyfluorescein diacetate (CFDA), and 2’,7’-dichlorodihydrofluorescein diacetate (DCFH-DA) to evaluate membrane potential, membrane integrity, metabolic activity, and ROS production, respectively, and were analyzed using flow cytometry (Figure 5 A).

**Figure 5:**
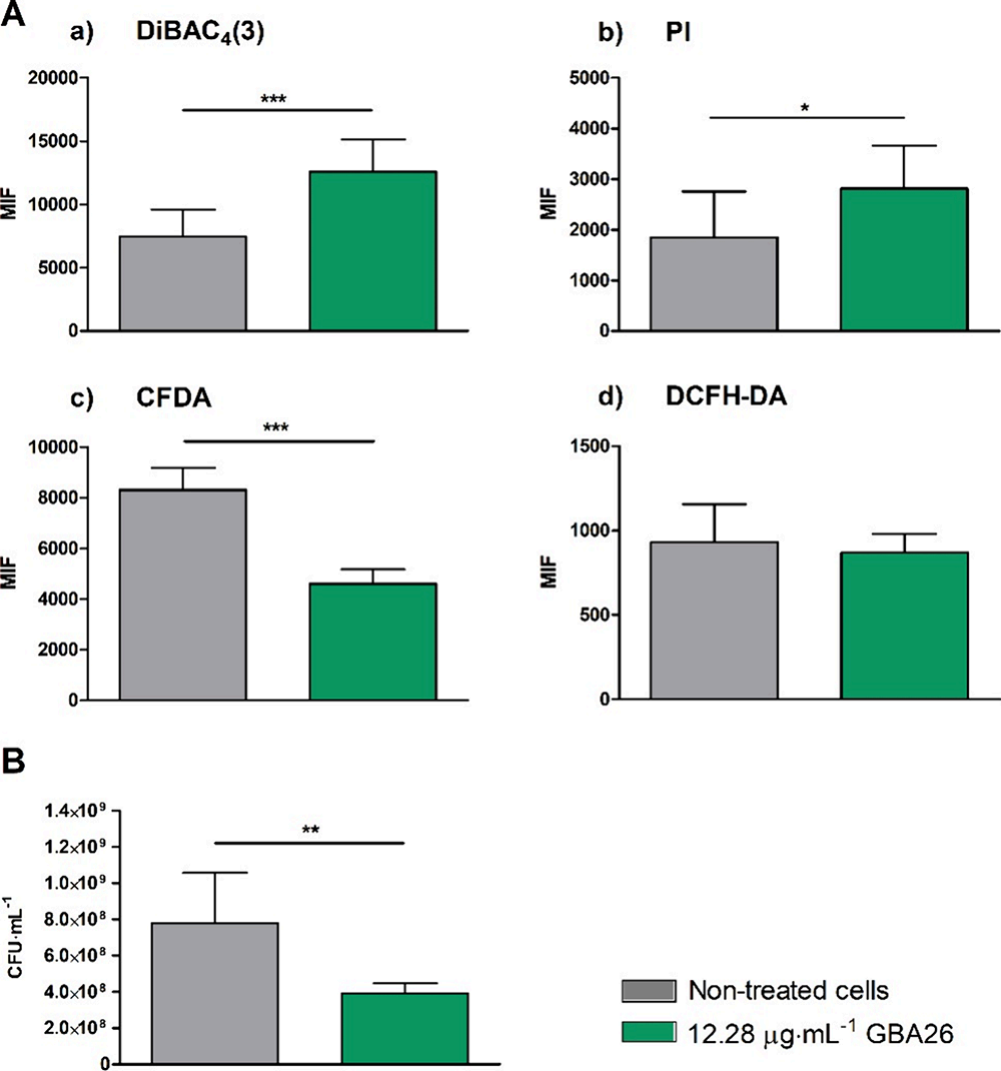
Effects of GBA26 on *Pseudoalteromonas tunicata* bacteria. (A) Mean intensity of fluorescence (MIF) of *P. tunicata* nontreated (gray) and treated with 12.28 μg·mL^–1^ of GBA26 (green) for 24 h. Cells were stained with (a) bis­(1,3-dibutylbarbituric acid) trimethine oxonol (DiBAC_4_(3)), (b) propidium iodide (PI), (c) 5(6)-carboxyfluorescein diacetate (CFDA), and (d) 2′,7′-dichlorodihydrofluorescein diacetate (DCFH-DA) and analyzed through flow cytometry. (B) Cell culturability of *P. tunicata* after exposure to the conditions described above. Results are presented as mean ± SD. Significant differences are indicated by *­(*p* < 0.05), **­(*p* < 0.01), and ***­(*p* < 0.001).

Flow cytometric data revealed that, under the tested conditions, GBA26 changes the cell membrane permeability of *P. tunicata*, as demonstrated by the cell staining with DiBAC_4_(3) and PI (Figure 5A-a and A-b). DiBAC_4_(3) enters depolarized cells, while PI intercalates the DNA of membrane-compromised cells, thereby increasing cell fluorescence.^56,57^ At the time of analysis, 10% of the cell population exposed to GBA26 exhibited alterations in membrane potential, resulting in a 1.69-fold increase in the MIF of treated cells compared to untreated cells (*p* < 0.001; Figure 5A-a). Furthermore, approximately 30% of the cell population experienced more extensive membrane damage, allowing PI to enter and resulting in a 1.52-fold increase in the MIF compared to untreated cells (*p* = 0.034; Figure 5A-b). These results suggest that (1) the compound induced depolarization of bacterial cell membranes, leading to pore formation; or (2) the compound caused membrane rupture, which in turn leads to nonspecific depolarization.^58^

CFDA staining showed that when *P. tunicata* is exposed to GBA26, approximately 40% of the cell population changed its metabolic activity. The analysis of MIF revealed that bacteria treated with GBA26 exhibited 1.80-fold lower MIF than the control (*p* < 0.001; Figure 5A-c), indicating that GBA26 reduced bacterial metabolic activity. CFDA is a nonfluorescent lipophilic substrate that is hydrolyzed by esterases in the cytoplasm, forming fluorescent carboxyfluorescein, and is used to assess metabolic activity.^59,60^ Nevertheless, the decrease in fluorescence intensity may also be associated with damage to the cell membranes (demonstrated by PI staining), which could lead to the leakage of the CFDA fluorescent product.^61^ The effect of GBA26 on bacterial culturability was also evaluated (Figure 5B). Data showed that, after 24 h of exposure to GBA26, the number of *P. tunicata* culturable cells decreased by 50% (*p* = 0.003) which can be explained by membrane damage, metabolic changes or other unidentified mechanisms. Gallic acid, the precursor of GBA26, demonstrated antibacterial activity against pathogenic bacteria, with a suggested mechanism of action related to the modification of cytoplasmic membrane function, among others.^62^ Gallic acid-induced permeation of the outer membrane was correlated with alterations in the inner membrane due to exposure to toxic agents, disruption of the electrochemical gradient, leakage of cellular components (e.g., DNA and proteins), disruption of ATP production, and the alteration of vital functions.^62^

Treated cells stained with DCFH-DA presented a similar MIF to untreated cells (Figure 5A-d), suggesting that, under the tested conditions, GBA26 did not induce the endogenous production of ROS. Oxidative stress is associated with cellular membrane alterations through the production of ROS.^63^ As such the observed membrane damage was not attributable to ROS production. Therefore, the antioxidant activity of GBA26 was subsequently tested.

### 3.3 GBA26 Antioxidant Properties

GBA26 is a derivative of gallic acid, a polyphenolic natural compound well-known for its antioxidant properties, due to the ability to scavange free radicals and chelate metal ions.^64^ The antioxidant properties of GBA26 were evaluated by 2,2-diphenyl-1-picrylhydrazyl (DPPH) radical scavenging capability and ferric reducing capacity (FRAP) assays and compared with antioxidant reference compounds gallic acid and Trolox. Notably, in the DPPH assay, GBA26 was the most active compound, exhibiting an EC_50_ value of 23.6 ± 0.01 μM lower than that of gallic acid (EC_50_ = 29.4 ± 0.05 μM) and Trolox (EC_50_ = 26.4 ± 0.01 μM). Regarding the FRAP assay, 1.7 ± 0.003 Trolox equivalents (eqs) were obtained for GBA26 versus a value of 2.5 ± 0.007 eqs obtained for gallic acid, also highlighting the antioxidant capability of GBA26.

## 4 Conclusions

Studying the interaction between bioactive compounds and biological membranes has become highly relevant in human health applications. Membranes are the interface with the external environment and act as a barrier for xenobiotics permeation into the organism/cell. However, few studies have been dedicated to the investigation of the interaction of antifouling agents with the biological membranes of target organisms as a putative mechanism of action for environmental applications. The aim of this work was to elucidate the interaction of GBA26 with bacterial membranes at the molecular and cellular levels in order to gain mechanistic insight into its antifouling mode of action. Although not intended as a screening methodology, the combined use of Langmuir monolayers with defined phospholipid and LPS compositions, together with complementary cellular assays, provides a framework for constructing simplified bacterial membrane model interfaces. These tunable models capture key physicochemical features of bacterial inner and outer membranes, such as surface charge, packing, and interfacial organization, and can be applied to probe membrane destabilization processes relevant to early stages of biofouling and biointerface formation. Within this work it was demonstrated that GBA26 can interact with phospholipids and LPS in different Langmuir monolayer membrane models mimicking bacterial inner and outer membranes. These findings were validated by studying GBA26 mechanism of action using live *P. tunicata* cells, revealing that GBA26 disrupts bacterial cell membranes. Furthermore, the disruption of membrane integrity by GBA26 seems to be associated with reduced metabolic activity and loss of cell culturability, without inducing oxidative stress. It is noteworthy that GBA26 has antioxidant properties due to its phenolic nature, as evidenced by DPPH and FRAP assays. Therefore, as ROS are not generated, lipid peroxidation does not occur, and the observed membrane damage is attributed to the direct interaction of GBA26 with the membrane, as evidenced by the Langmuir lipid monolayer studies at the molecular level. Altogether, the present work sheds light on the antibiofilm mechanism of action of GBA26, as an antifouling agent, by interfering with bacterial membranes.

## Supplementary Material



## Notes

Surface pressure–area isotherms of pure DPPE and DPPG monolayers; independent replicates of pressure–area isotherms for each tested monolayer and condition; table of DPPE and DPPG monolayers’ parameters (Cs^1–^_max_, A_0_, A_10_, A_30_); tables of experimentally derived, ideal, and excess molecular areas for binary and ternary lipid mixtures; MALDI-TOF spectra of LPS from *P. aeruginosa* and *P. tunicata*; and BAM images of LPS from *P. aeruginosa* monolayer at different surface pressures (PDF)
