# Supplementary material for: Surface Interactions between an Eco-Friendly Antifouling Agent and Pseudoalteromonas tunicata Membrane
Source: ACS Appl Bio Mater. 2026 Mar 6;9(6):3109–21. doi: 10.1021/acsabm.5c02341 (PMC12997153; doi:10.1021/acsabm.5c02341)
Supplement: Supplementary file 1 [file mt5c02341_si_001.pdf]

Supporting Information

Surface interactions between an eco-friendly antifouling agent and *Pseudoalteromonas tunicata* membrane

Ana Sara Gomes[1,2*]; Cláudia Nunes[3]; Rita Teixeira-Santos[4,5]; Maria Romeu[4,5]; Maria Laura Alfieri[6]; Sara M. M. Cravo[2]; Filipe Mergulhão[4,5]; Marta Correia-da-Silva[1,2*]; Salette Reis[3]

1 CIIMAR/CIMAR LA, Interdisciplinary Centre of Marine and Environmental Research, University of Porto, Terminal de Cruzeiros do Porto de Leixões, 4450-208 Matosinhos, Portugal.

2 Laboratory of Organic and Pharmaceutical Chemistry, Faculty of Pharmacy, University of Porto, Rua Jorge de Viterbo Ferreira 228, 4050-313 Porto, Portugal.

3 LAQV, REQUIMTE, Departamento de Ciências Químicas, Faculdade de Farmácia, Universidade do Porto, Rua Jorge Viterbo Ferreira 228, 4050-313, Porto, Portugal.

4 LEPABE-Laboratory for Process Engineering, Environment, Biotechnology and Energy, Faculty of Engineering, University of Porto, Rua Dr. Roberto Frias, 4200-465 Porto, Portugal.

5 ALiCE-Associate Laboratory in Chemical Engineering, Faculty of Engineering, University of Porto, Rua Dr. Roberto Frias, 4200-465 Porto, Portugal.

6 Department of Chemical Sciences, University of Naples Federico II, Via Cintia 4, I-80126, Naples, Italy.

*sara.gomes@ciimar.up.pt; m_correiadasilva@ff.up.pt

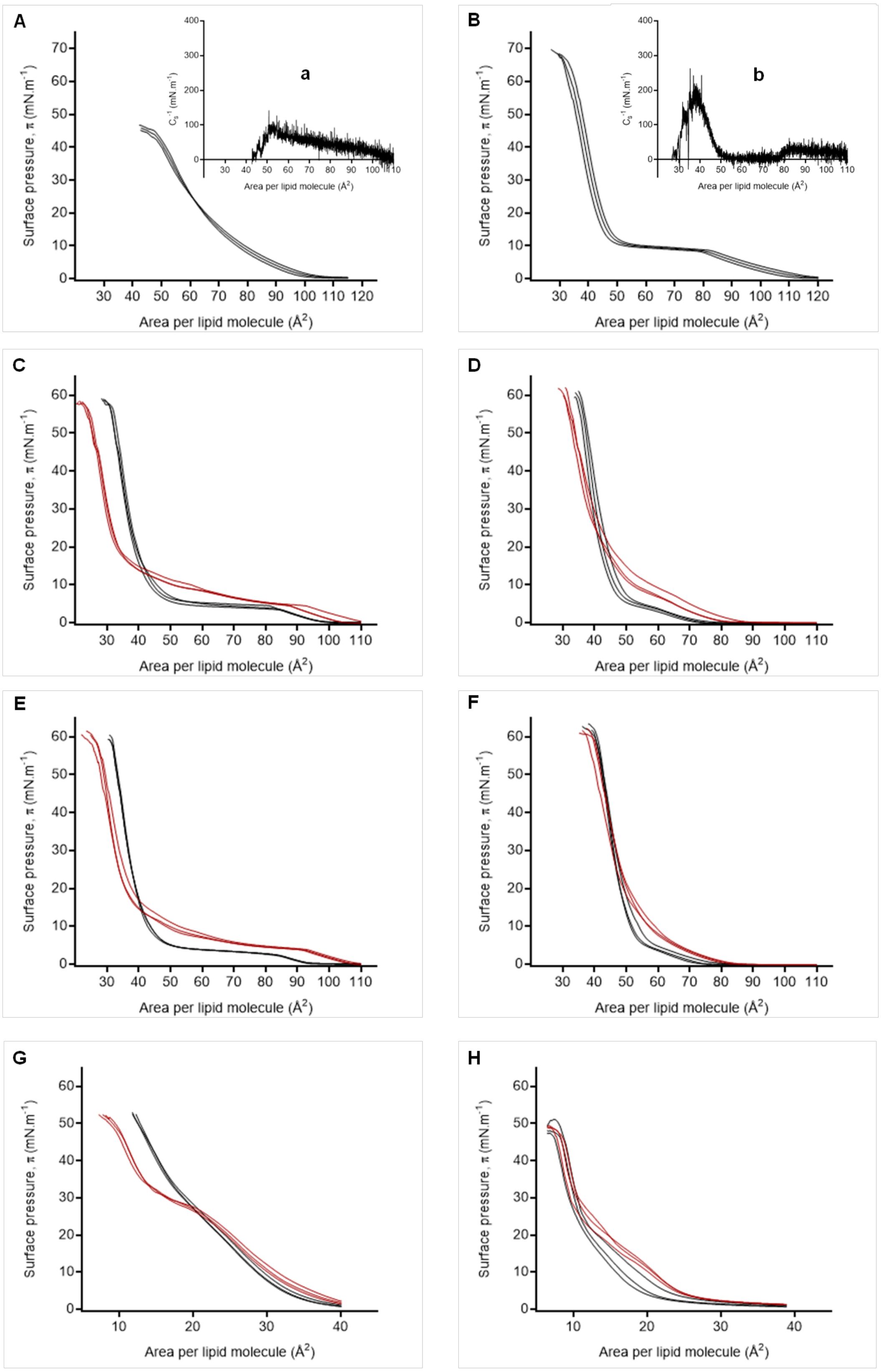

Figure S1. Surface pressure-area isotherms of pure and mixed lipid monolayers: (**A**) 100% DPPE, (**B**) 100% DPPG, (**C**) 100% DPPC, (**D**) DPPC:DPPE (7:3), (**E**) DPPC:DPPG (9:1), (**F**) DPPC:DPPE:DPPG (6:3:1), (**G**) 100% LPS from *Pseudomonas aeruginosa*, and (**H**) 100% LPS extracted from *Pseudoalteromonas tunicata*. Isotherms were recorded on 10 mM HEPES containing 100 mM NaCl (pH 7.4) at 21 °C, in the absence (black) or presence (red) of GBA26 (50 µM). For each condition, three independent replicate isotherms are shown. (**a**) and (**b**) insets are the compressibility modulus (Cs^{-1}) graphs corresponding to one of the isotherms represented.

Table S1. Isotherm-derived values of the areas per lipid molecule and the maximum compressibility modulus for pure DPPC, DPPG and DPPE monolayers.

Pure monolayer	$C_s^{-1}{}_{max}$ (mN·m^{-1})	A_0 (Å^2)	A_{10} (Å^2)	A_{30} (Å^2)
**DPPC**	200 ± 3	41.8 ± 0.5	53.2 ± 1.9	42.6 ± 0.4
**DPPE**	92 ± 3	73.8 ± 0.8	94.0 ± 2.4	76.8 ± 1.2
**DPPG**	187 ± 4	43.7 ± 0.2	59.3 ± 2.1	48.2 ± 1.4

$C_s^{-1}{}_{max}$: maximum compressibility modulus; A_0: minimum area per lipid molecule; A_{10}: area per lipid molecule at 10 mN·m^{-1} surface pressure; A_{30}: area per lipid molecule at 30 mN·m^{-1} surface pressure.

Table S2. Experimental molecular areas, ideal additivity values, and excess areas of mixed lipid monolayers at selected surface pressures (π = 0, 10, and 30 mN·m^{-1}).

	Mixed monolayer	A_0 (Å^2)	A_{10} (Å^2)	A_{30} (Å^2)
**Experimentally-derived mean values**	DPPC:DPPE (7:3)	45.2	52.0	45.9
	DPPC:DPPG (9:1)	42.2	49.9	42.4
	DPPC:DPPE:DPPG (6:3:1)	51.7	57.7	51.9
**Ideal additivity values**	DPPC:DPPE (7:3)	51.4	65.4	52.9
	DPPC:DPPG (9:1)	42.0	53.8	43.2
	DPPC:DPPE:DPPG (6:3:1)	51.6	66.05	53.4
**Excess area (A_{exc})**	DPPC:DPPE (7:3)	-6.2	-13.4	-7.0
	DPPC:DPPG (9:1)	0.2	-3.9	-0.3
	DPPC:DPPE:DPPG (6:3:1)	0.11	-8.4	-1.5

A_0: minimum area per lipid molecule; A_{10}: area per lipid molecule at 10 mN·m^{-1} surface pressure; A_{30}: area per lipid molecule at 30 mN·m^{-1} surface pressure.

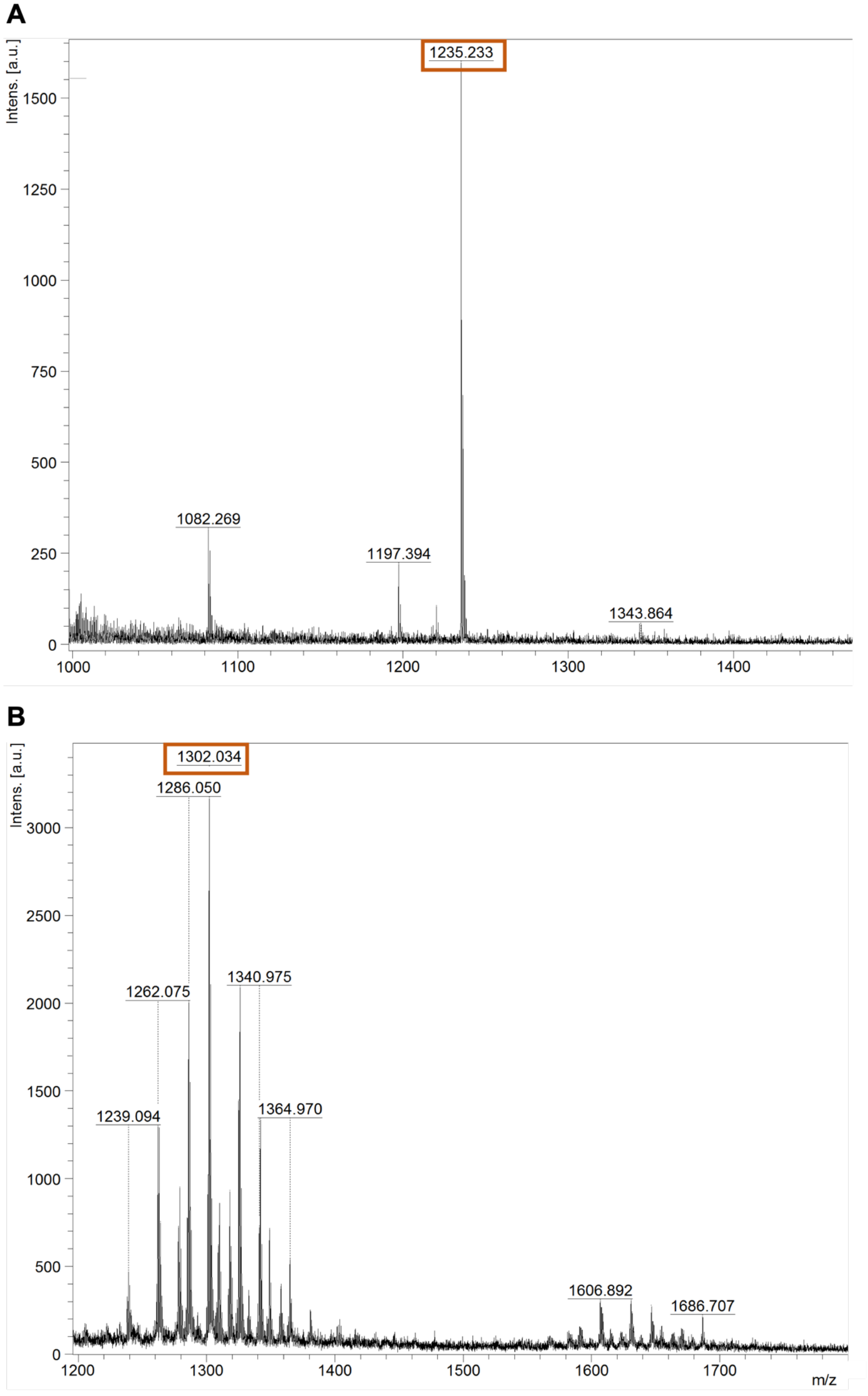

Figure S2. Zoom of the reflectron MALDI-TOF spectra in negative-ion mode of lipid A of LPS from (**A**) *Pseudoalteromonas tunicata* and (**B**) *Pseudomonas aeruginosa*. Orange square highlights the most abundant species.

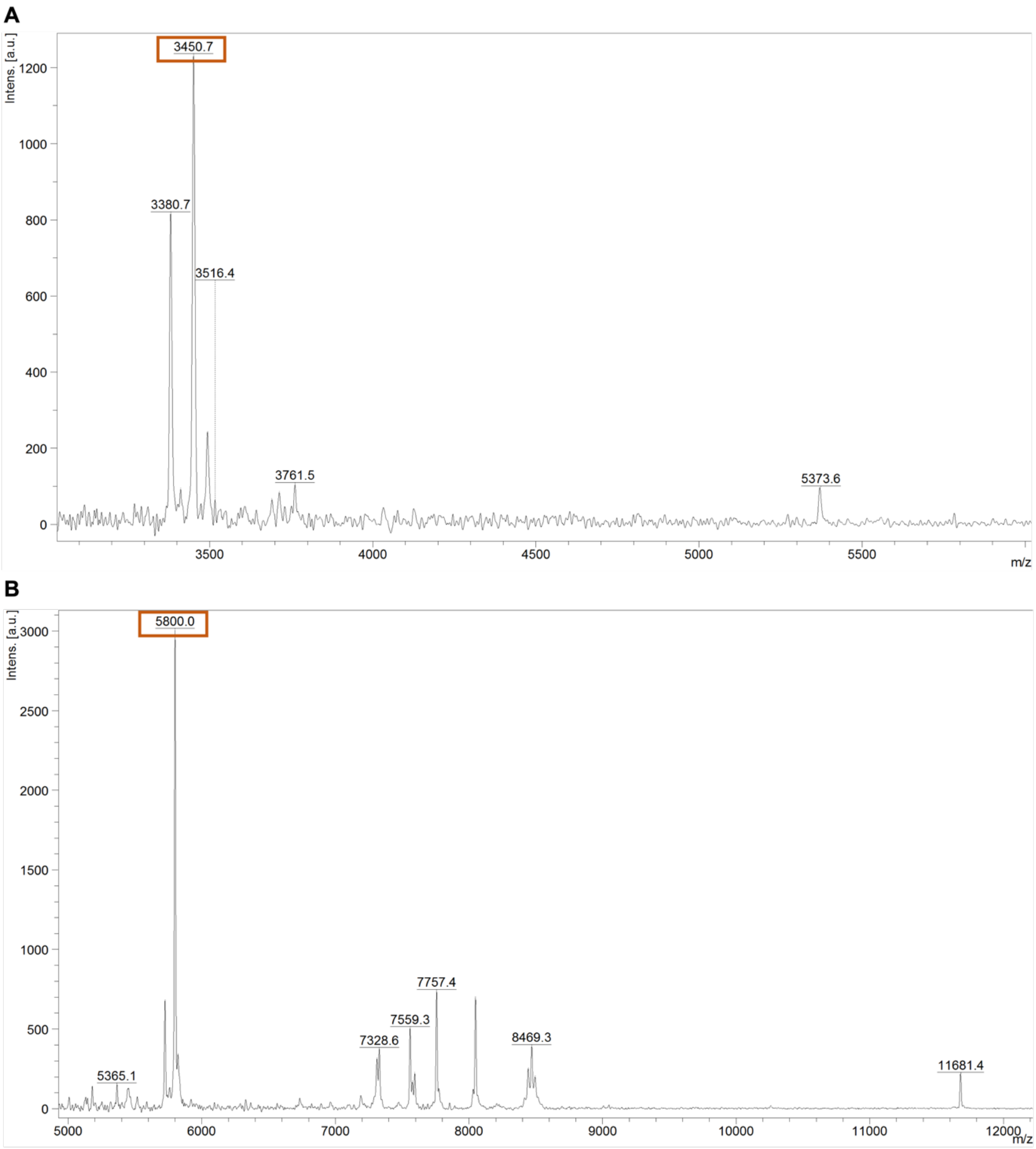

Figure S3. Zoom of the linear MALDI-TOF spectra in positive-ion mode of LPS from (**A**) *Pseudoalteromonas tunicata* and (**B**) *Pseudomonas aeruginosa*. Orange square highlights the most abundant species.

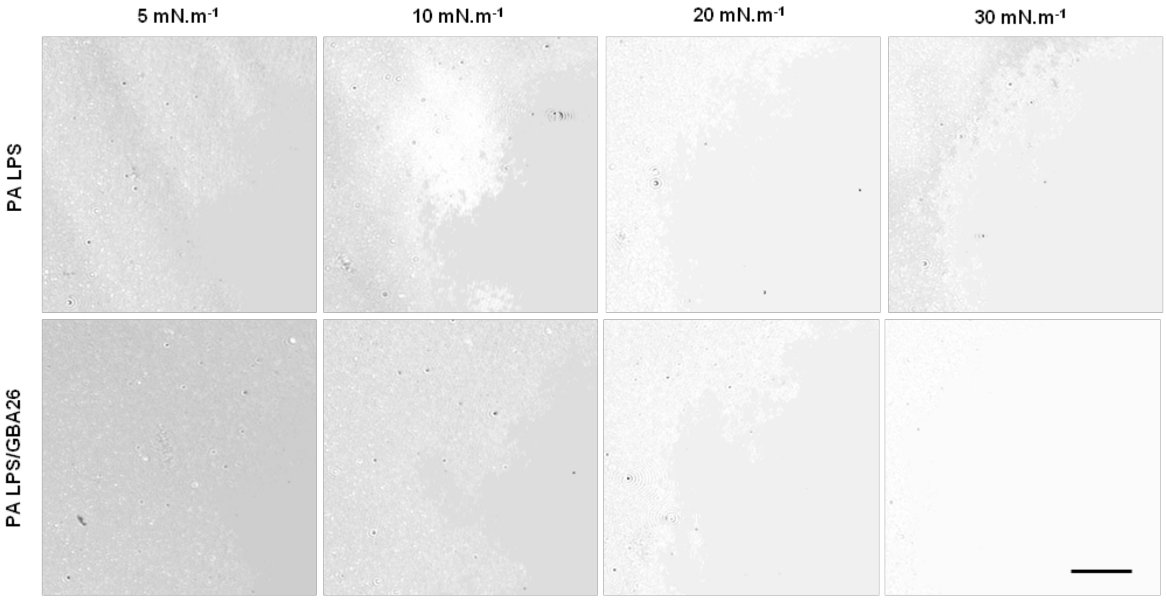

Figure S4. LPS forms a condensed, film-like monolayer with no observable lipid domains. Brewster angle microscopic images of Langmuir monolayers of lipopolysaccharide from *Pseudomonas aeruginosa* (PA LPS), at 5, 10, 20, 30 mN·m^{-1} pressures on 10 mM HEPES, 100 mM NaCl (pH 7.4) at 21° C. The top and bottom rows, of each set, correspond to the monolayer on the subphase without and with GBA26 at 50 µM, respectively. Scale bar represents 100 µm.
